# Supplementary material for: Maintenance of variation in virulence and reproduction in populations of an agricultural plant pathogen
Source: Evol Appl. 2020 Sep 24;14(2):335–47. doi: 10.1111/eva.13117 (PMC7896723; doi:10.1111/eva.13117)
Supplement: Supplementary file 1 — Supplementary Material [file EVA-14-335-s001.docx]

**Supplemental Table 1**. Summary statistics of virulence (amount of necrotic lesion area) and reproduction (pycnidia density within lesions) in each host and population from 145 *Zymoseptoria tritici* isolates using the non-transformed data.

|  | Virulence | | | | | Reproduction | | |
| --- | --- | --- | --- | --- | --- | --- | --- | --- |
|  |  | Mean | Range | SE* |  | Mean | Range | SE |
| Host | 1011 | 36.8 | 0-100 | 2.5 |  | 3.2 | 0-79 | 0.6 |
|  | 1204 | 77.3 | 0-100 | 1.4 |  | 70.0 | 0-671 | 4.3 |
|  | 4391 | 85.7 | 2.7-100 | 1.1 |  | 87.8 | 0-588 | 3.9 |
|  | 5254 | 49.9 | 0-100 | 2.1 |  | 14.1 | 0-332 | 1.7 |
|  | ArinaLr34 | 57.9 | 0-100 | 1.4 |  | 20.7 | 0-382 | 1.4 |
|  | Chinese Spring | 78.2 | 0-100 | 1.0 |  | 22.5 | 0-538 | 1.3 |
|  | Drifter | 68.3 | 0-100 | 1.1 |  | 20.0 | 0-418 | 1.0 |
|  | Gene | 38.9 | 0-100 | 1.0 |  | 4.1 | 0-200 | 0.3 |
|  | Greina | 60.7 | 0-100 | 1.0 |  | 8.1 | 0-205 | 0.5 |
|  | Runal | 59.7 | 0-100 | 0.9 |  | 10.9 | 0-220 | 0.5 |
|  | Titlis | 75.5 | 0-100 | 0.8 |  | 3.5 | 0-92.3 | 0.3 |
|  | Toronit | 42.2 | 0-100 | 1.1 |  | 3.1 | 0-74.8 | 0.2 |
| Population | Australia | 50.1 | 0-100 | 0.8 |  | 6.72 | 0-275 | 0.4 |
|  | Israel | 56.2 | 0-100 | 0.8 |  | 33.9 | 0-671 | 1.5 |
|  | Oregon.R | 70.0 | 0-100 | 0.7 |  | 14.2 | 0-426 | 0.6 |
|  | Oregon.S | 66.5 | 0-100 | 0.8 |  | 20.7 | 0-588 | 1.1 |
|  | Switzerland | 63.8 | 0-100 | 0.8 |  | 13.1 | 0-382 | 0.6 |

*SE= Standard Error

**A**

**B**

**Supplemental Figure 1**. Frequency distribution of (A) virulence (amount of necrotic lesion area) and (B) reproduction (pycnidia density within lesions) among 145 *Zymoseptoria tritici* isolates from five populations in 12 hosts. Data were log-transformed.

**A**

**B**

**D**

**C**

**Supplemental Figure 2**. Correlation between (A) overall mean virulence (amount of necrotic lesion area) and variance of virulence, (B) overall mean reproduction and variance of reproduction, (C) overall mean reproduction (pycnidia density within lesions) and standard coefficient of variation for reproduction, and (D) maximum virulence and reproduction specialization among 145 *Zymoseptoria tritici* isolates from five populations. Specialization represents the estimates of adjusted coefficient of variation of means across 12 hosts for each trait. Higher specialization indicates preference for specific hosts to maximize trait performance. Data were log-transformed.

**B**

**A**

**C**

**Supplemental Figure 3**. Boxplot showing the distribution of (A) overall mean virulence (amount of necrotic lesion area), (B) reproduction (pycnidia density within lesions) specialization, and (C) maximum reproduction among 145 *Zymoseptoria tritici* isolates from five populations in 12 hosts. Specialization represents the estimates of adjusted coefficient of variation of means across 12 hosts for each trait. Higher specialization indicates preference for specific hosts to maximize trait performance. Data were log- transformed.
